# 

**Published:** 1881-09

**Authors:** 


ESTABLISHED IN 1855.

### NEW ARRANGEMENT

It is with pleasure the Proprietor of the old and well known

### Atlanta Medical and Surgical Journal,

Announces to the Medical Profession that he has made an entirely new arrangement in

**THE EDITORIAL MANAGEMENT**

Of the JOURNAL, and one which will, no doubt, meet the approval and receive the support of the profession in the Southern States.

The Editorial Department of the JOURNAL will, in future, be under the direction of DR. JAMES B. BAIRD and DR. JNO. THAD. JOHNSON.

These gentlemen are already well known to the medical profession; and, being comparatively young and possessing a taste for journalistic work, will give their time and talents to making the ATLANTA MEDICAL AND SURGICAL JOURNAL what it should be—the leading Medical Journal of the South Atlantic States.

Without disparagement to the former editors, it is patent that the JOURNAL has not, for the past year or two, reached the full measure of a progressive medical periodical. In the future we shall endeavor to place it in the front rank and to make it the exponent of the views of the best medical minds of the South. The JOURNAL will not be the organ of any individual, institution or organization: It will be purely independent, and conducted solely in the interests of its readers.

The editors have already secured promises from some of our best practitioners to contribute to its pages during the coming year. The names of the contributors will be subsequently announced. For the present suffice it to say that these gentlemen will give to its pages an interest it has not possessed for many years.

Drs. Baird and Johnson will assume editorial control of the JOURNAL with the

**OCTOBER NUMBER, 1881,**

And any communication desired to appear after the 1st September should be addressed to either of them.

### REDUCTION OF PRICE.

Desiring to place the JOURNAL within the reach of the entire profession, it has been decided to reduce the price to $2.50 per year, and putting the price so low we cannot afford to "carry" any subscriptions. Our friends will therefore save us embarrassment by remitting the money with their orders for the JOURNAL, as we shall be compelled to decline sending the JOURNAL to any address unless the order is accompanied by the cash. We shall publish a good journal, one well worth the price, and shall expect to receive the money for every one sent out.

The JOURNAL will still contain 64 pages of reading matter, and will be printed in large, plain type and on clear, white paper, securely bound and neatly trimmed.

### CLUB RATES.

To any Physician who will send us five names and $12.50 we will send one extra copy of the JOURNAL free for one year.

Address all communications in regard to subscriptions, advertisements or any other business matter to H. H. DICKSON, Publisher, 32 Broad Street. Any communication intended for publication should be addressed to either of the editors.

### A SPECIAL OFFER.

Any subscriber remitting before the 1st of September will receive the September number free.

### SAMPLE COPIES

Of the October number will be furnished free to any Physician who will send us his name before the 20th of September; after that time we will send sample copies on receipt of 25 cents.

☞ The October number will be ready for issue on or about the 20th of September.

H. H. DICKSON,
**32 Broad St., Atlanta, Ga.**

